# Explainable AI-Driven Quality and Condition Monitoring in Smart Manufacturing

**DOI:** 10.3390/s26030911

**Published:** 2026-01-30

**Authors:** M. Nadeem Ahangar, Z. A. Farhat, Aparajithan Sivanathan, N. Ketheesram, S. Kaur

**Affiliations:** 1 AMRC North West, University of Sheffield, Blackburn BB2 7HN, UK; z.farhat@amrc.co.uk (Z.A.F.); a.sivanathan@amrc.co.uk (A.S.); k.nilanesan@amrc.co.uk (N.K.); 2 School of Mechanical, Aerospace and Civil Engineering, University of Sheffield, Mappin Street, Sheffield S1 4DT, UK; skaur7@sheffield.ac.uk

**Keywords:** explainable artificial intelligence (XAI), trustworthy AI, industrial AI, smart manufacturing, visual inspection, acoustic anomaly detection, human-in-the-loop systems, predictive maintenance, SHAP, Grad-CAM, Industry 5.0

## Abstract

Artificial intelligence (AI) is increasingly adopted in manufacturing for tasks such as automated inspection, predictive maintenance, and condition monitoring. However, the opaque, black-box nature of many AI models remains a major barrier to industrial trust, acceptance, and regulatory compliance. This study investigates how explainable artificial intelligence (XAI) techniques can be used to systematically open and interpret the internal reasoning of AI systems commonly deployed in manufacturing, rather than to optimise or compare model performance. A unified explainability-centred framework is proposed and applied across three representative manufacturing use cases encompassing heterogeneous data modalities and learning paradigms: vision-based classification of casting defects, vision-based localisation of metal surface defects, and unsupervised acoustic anomaly detection for machine condition monitoring. Diverse models are intentionally employed as representative black-box decision-makers to evaluate whether XAI methods can provide consistent, physically meaningful explanations independent of model architecture, task formulation, or supervision strategy. A range of established XAI techniques, including Grad-CAM, Integrated Gradients, Saliency Maps, Occlusion Sensitivity, and SHAP, are applied to expose model attention, feature relevance, and decision drivers across visual and acoustic domains. The results demonstrate that XAI enables alignment between model behaviour and physically interpretable defect and fault mechanisms, supporting transparent, auditable, and human-interpretable decision-making. By positioning explainability as a core operational requirement rather than a post hoc visual aid, this work contributes a cross-modal framework for trustworthy AI in manufacturing, aligned with Industry 5.0 principles, human-in-the-loop oversight, and emerging expectations for transparent and accountable industrial AI systems.

## 1. Introduction

AI and machine learning (ML) are increasingly reshaping industrial domains by delivering high-performing, data-driven solutions such as automated defect detection, predictive maintenance, and process optimisation. Deep learning, in particular, has achieved state-of-the-art results in areas such as computer vision and signal analysis [1,2,3]. Over the past decade, AI has evolved into a transformative technology that is redefining industrial systems, particularly within manufacturing. Its ability to process vast amounts of heterogeneous data has enabled unprecedented levels of automation, optimisation, and predictive decision-making. However, the same complexity that drives its success especially in deep learning models with millions of parameters has raised significant concerns about trust, transparency, and accountability in safety-critical environments. As industries integrate AI into production systems, ensuring trustworthiness becomes a prerequisite rather than an afterthought, since decision-making in manufacturing must be both accurate and interpretable.

The widespread adoption of AI has resulted in complex socio-technical systems where rapid, automated decision-making often replaces slower traditional analyses. While efficient, this has raised concerns about users placing excessive trust in AI outputs. Misplaced trust can lead to over-reliance, with users accepting AI recommendations uncritically [4]. Such reliance risks treating embedded biases or errors, including AI hallucinations, as objective truth without adequate validation.

Studies further indicate that excessive reliance on AI can diminish users’ creativity, critical thinking, and practical skills [5,6,7]. In manufacturing, although AI can process large volumes of data to support decisions, the underlying meaning may be lost without human contextual understanding. Over-reliant users may perform well in familiar situations but struggle in novel scenarios where prior data is unavailable, limiting their ability to reason, adapt, and generate actionable insights.

Bias in AI systems can arise from multiple sources, including user, data, and algorithmic bias [8]. Over-reliance on AI makes such biases difficult to detect, as outputs are often perceived as inherently trustworthy, potentially reinforcing existing prejudices [8,9]. These issues have led to documented discriminatory outcomes in socio-technical systems, notably in criminal justice [10] and healthcare [11].

Despite these challenges, users continue to adopt AI due to its efficiency and rapid decision-making capabilities [4]. However, excessive reliance poses long-term risks such as cognitive offloading, skill degradation, and reduced accountability. A balanced approach is therefore required, where AI supports decision-making while humans retain understanding and responsibility. This necessitates transparency and explainability, enabling users to understand not only what decisions are made, but why. As manufacturing and other critical sectors increasingly depend on AI, trustworthiness grounded in explainability, accuracy, and interpretability becomes essential for maintaining human-centred control over decisions.

To bridge this gap, XAI has emerged as a paradigm that renders model reasoning transparent to engineers, regulators, and operators. In manufacturing, where data are generated from diverse sources including, but not limited to, visual imagery, acoustic signals, and multivariate sensor data, explainability ensures that algorithmic decisions remain auditable and physically meaningful. Incorporating XAI therefore becomes essential not only for compliance [12] but also for actionable insights that enhance reliability and reduce downtime.

Beyond performance, industrial AI must also be trustworthy. According to the European Commission’s High-Level Expert Group on AI (HLEG), a trustworthy system must be lawful, ethical, and robust throughout its lifecycle [13]. Among its seven key requirements, transparency and human-in-the-loop (HITL) oversight are especially relevant to manufacturing, where engineers must interpret, validate, or override model outputs before deployment [14]. This human-centric perspective aligns with the transition from Industry 4.0—automation-driven—to Industry 5.0, which re-centres human creativity and collaboration [15,16]. Within this paradigm, AI acts as a partner that augments human capability, extending trustworthiness to ethical and operational dimensions. Building upon this foundation, Table 1 summarises the principal forms of manufacturing data and their corresponding XAI use cases. These modalities—vision, acoustic/vibration, and multivariate sensor data—capture complementary aspects of industrial processes. To explore explainability across them, the present work examines three representative datasets: Casting Product (image classification), Defects Class and Location (object detection), and MIMII (acoustic anomaly detection). Each dataset is evaluated independently to illustrate how XAI techniques contribute to improved transparency and diagnostic understanding.

While significant advances have been made in applying deep learning models to manufacturing tasks such as visual inspection and condition monitoring, their widespread adoption remains constrained by the opaque, black-box nature of these systems. In industrial environments particularly those that are safety-critical, quality-sensitive, or regulated the ability to understand and audit AI-driven decisions is as important as predictive accuracy.

The primary objective of this paper is not to propose or optimise new machine learning models, but to systematically investigate how XAI techniques can be used to open the black-box behaviour of AI systems commonly encountered in manufacturing. To this end, the study deliberately employs multiple representative models across heterogeneous data modalities—vision-based classification, vision-based detection, and acoustic anomaly detection to examine whether XAI methods can provide consistent, physically meaningful explanations irrespective of model architecture or learning paradigm. The key contributions of this work are as follows:

- Cross-modal explainable AI framework: The paper presents a unified interpretive approach for applying explainable AI techniques across heterogeneous manufacturing data modalities, encompassing supervised vision-based classification, vision-based defect localisation, and unsupervised acoustic anomaly detection. This demonstrates the generalisability of XAI techniques beyond a single task, model architecture, or learning paradigm. This approach focuses on structured interpretation of XAI outputs rather than the development of quantitative explainability metrics.
- Physically grounded interpretation of AI decisions: The study systematically links XAI outputs to physically meaningful defect characteristics and fault mechanisms, enabling domain-grounded interpretation of model behavior rather than purely visual or statistical explanation. This supports actionable insight and diagnostic reasoning in industrial environments.
- Explainability as a trust-enabling mechanism: The work positions explainability as an operational component of trustworthy AI, aligned with Industry 5.0 principles, human-in-the-loop oversight, and emerging regulatory expectations for transparency and accountability in manufacturing AI systems.

In this work, systemising explainable AI refers to the structured and repeatable integration of explainability into heterogeneous industrial AI pipelines, where explainability is treated as a primary analytical component rather than a post hoc visualisation. By focusing on interpretability, transparency, and trust rather than model optimisation, this work bridges the gap between theoretical XAI research and the practical demands of real-world industrial deployment through a systemised integration of explainability across heterogeneous manufacturing applications. In this context, trustworthiness is addressed through interpretability and design alignment with Industry 5.0 principles, while the empirical evaluation of operator trust or empowerment through user studies is beyond the scope of this work.

The remainder of this paper is organised as follows. Section 2 provides the theoretical foundation of trustworthy and XAI in industrial contexts, emphasising the role of human oversight, transparency, and ethical governance frameworks relevant to Industry 5.0. Similarly, Section 3 discusses relevant prior studies and contributions from the existing literature. Section 4 reviews benchmark datasets used in industrial defect detection and condition monitoring, highlighting their relevance to AI trustworthiness and reproducibility. Section 5 examines the role of XAI in manufacturing, focusing on its contribution to transparency across visual inspection and sensor-based monitoring systems. Section 6 describes the proposed methodology, detailing data preprocessing, model training, and integration of explainability methods for visual and acoustic modalities. Section 7 presents and discusses the results, demonstrating how XAI enhances interpretability, diagnostic clarity, and operator trust in AI-driven manufacturing systems. Finally, Section 8 concludes the paper and outlines future research directions for advancing trustworthy and explainable AI within smart manufacturing ecosystems.

## 2. Background

As discussed in the Introduction, this section provides a detailed overview of the principles of trustworthy AI, the role of human oversight in Industry 5.0, and the importance of XAI in ensuring transparency and accountability within industrial systems.

**Figure 1:**
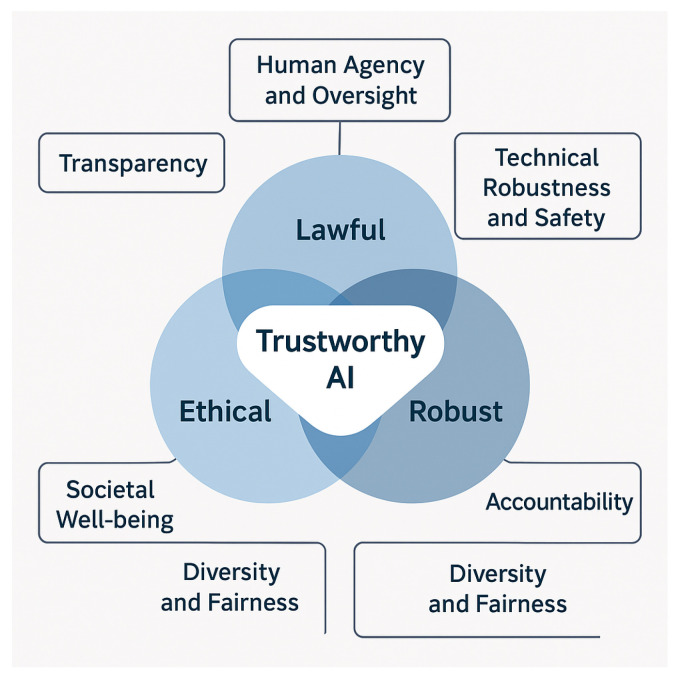
The three pillars and seven key requirements of Trustworthy AI, adapted from the European Commission’s HLEG framework [13]. These dimensions collectively define the foundation for ethical, lawful, and robust AI in industrial systems.

According to the European Commission’s High-Level Expert Group on Artificial Intelligence (HLEG), a trustworthy AI system must satisfy three interdependent components—it must be lawful, ethical, and robust throughout its lifecycle [13]. The HLEG further outlines seven key requirements for trustworthy AI: human agency and oversight, technical robustness and safety, privacy and data governance, transparency, diversity and fairness, societal well-being, and accountability. These principles collectively establish a foundation for deploying AI responsibly across industrial domains [20,21]. The overall structure of trustworthy AI, as proposed by the European Commission’s HLEG, is illustrated in Figure 1, highlighting the interrelation between the three pillars—lawfulness, ethics, and robustness and the seven key requirements that underpin responsible AI adoption.

**Figure 2:**
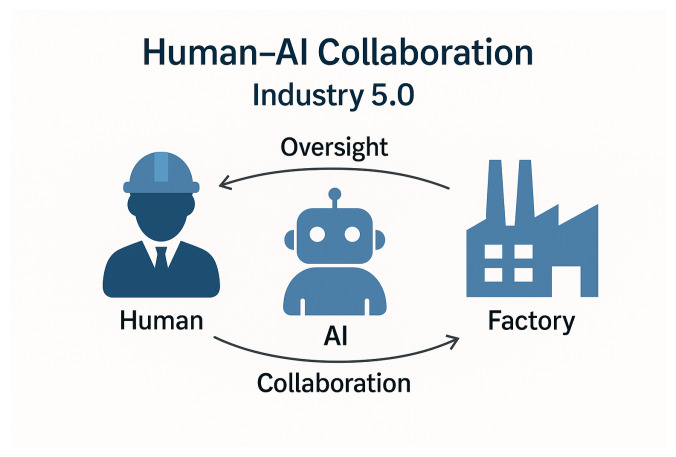
Human–AI collaboration within the Industry 5.0 paradigm, emphasising human oversight, validation, and collaboration between operators, AI systems, and industrial processes [15,22].

Among these pillars, transparency and human-in-the-loop (HITL) oversight are central to building operational trust. Transparency refers to the degree to which stakeholders including developers, operators, and regulators can understand an AI system’s data sources, decision processes, and limitations. The EU AI Act further formalises this requirement by mandating explainability and traceability for high-risk AI systems in industrial settings [14]. Complementarily, human-in-the-loop approaches ensure that AI systems augment rather than replace human decision-making, embedding oversight mechanisms that allow humans to interpret, validate, or override AI outputs when necessary. This human-centric perspective is essential in manufacturing, where system reliability, worker safety, and quality control depend on the interplay between human expertise and automated intelligence [22]. The central role of human oversight and collaboration within Industry 5.0 is illustrated in Figure 2, where humans, AI systems, and industrial processes operate in a continuous feedback loop that ensures transparency, validation, and shared control.

This emphasis on human–machine collaboration is reinforced by the ongoing transition from Industry 4.0 to Industry 5.0, a paradigm that places humans back at the core of industrial production. Industry 5.0 emphasises sustainability, resilience, and human-centric design over purely efficiency-driven automation. It integrates digital technologies—such as AI, the Internet of Things (IoT), robotics, and cyber-physical systems with human creativity and oversight [15]. Within this framework, AI becomes not only a tool for automation but also a partner in augmenting human capability. Consequently, the concept of trustworthiness expands to encompass ethical, social, and operational dimensions ensuring that intelligent manufacturing remains both productive and human-centred [16,23].

AI’s role in manufacturing has become particularly prominent in quality control, predictive maintenance, and process optimisation, where data-driven methods have achieved unprecedented accuracy. Deep learning models now power visual inspection systems for surface defect detection, vibration-based fault diagnosis, and multivariate sensor fusion for predictive maintenance. However, these models often operate as “black boxes,” providing results without interpretable reasoning. This opacity limits operator trust and hinders root-cause analysis when failures occur [24]. To mitigate such limitations, XAI has emerged as a critical approach to make AI reasoning comprehensible to engineers and auditors. By visualising which features or data regions influence predictions, XAI allows stakeholders to assess not just whether a model works, but why it produces a given outcome [25].

**Figure 3:**
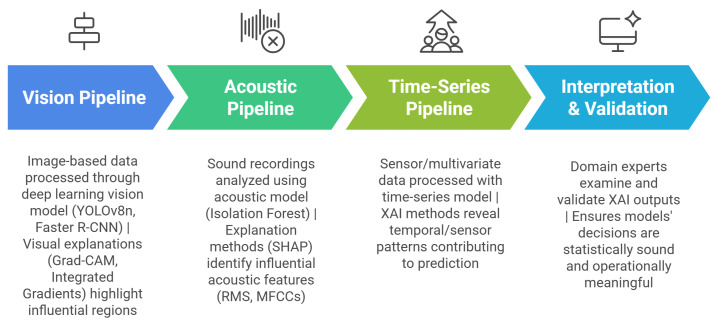
Overview of the three independent XAI pipelines used in this study. Vision, acoustic, and time-series data are each processed through dedicated AI models and corresponding explainability methods, with results evaluated in an Interpretation & Validation stage to ensure transparency and trust in manufacturing AI systems.

Explainability is particularly important when AI models interact with diverse data sources for instance, vision data from industrial cameras, acoustic signals from machine sensors, and multivariate time-series data from process monitoring systems. Each of these data domains captures complementary aspects of manufacturing behavior: visual data reveal surface and geometric anomalies; acoustic and vibration signals expose internal mechanical conditions; and sensor data track thermal, electrical, or dynamic system states. In this context, XAI provides mechanisms to interpret and validate model decisions across these modalities. For example, visual methods such as Grad-CAM or Saliency maps highlight spatial regions driving defect detection and classification, while SHAP (SHapley Additive exPlanations) explain feature-level contributions in time-series or acoustic analysis [26]. Such interpretability enables informed decision-making, facilitates troubleshooting, and reinforces user trust in AI-assisted manufacturing. The integration of XAI into the manufacturing pipeline is summarised in Figure 3, which depicts how data from visual, acoustic, and sensor sources feed into AI models, followed by explainability layers that generate interpretable outputs for human validation.

However, the deployment of AI without proper oversight has also led to notable failures and incidents. Studies on trustworthy AI have documented several cases where opaque or poorly validated AI models caused operational disruptions, safety risks, or biased outcomes [27]. These “AI disasters” underscore the importance of embedding risk management and governance frameworks such as ISO/IEC 23894:2023 [28] and the NIST AI RMF 1.0, both of which provide structured methodologies for managing AI reliability, bias, and transparency. In contrast, successful AI deployments in manufacturing, those integrating explainability, human supervision, and compliance with standards demonstrate measurable improvements in productivity, defect detection, and worker confidence [23]. These examples reveal that trustworthy AI is not merely a regulatory ideal but a tangible enabler of industrial excellence.

A growing body of related work now explores how explainability, transparency, and accountability can be quantitatively evaluated in industrial AI systems. Frameworks such as BEExAI [29], OpenHEXAI [30], and OpenXAI benchmark explainability methods across metrics like fidelity, stability, and human interpretability [31]. Recent surveys emphasise the need for domain-specific adaptations of these toolkits for manufacturing, where real-time decision-making, physical safety, and multimodal data integration present unique challenges [27]. Establishing objective criteria for evaluating XAI in manufacturing is thus a vital step toward operationalising trustworthy AI principles.

Building upon this conceptual foundation, the present study investigates how explainable AI techniques can enhance transparency and reliability across manufacturing domains characterised by visual, acoustic, and sensor-based data. By applying interpretable models and visualisation methods to representative datasets, this work aims to demonstrate how explainability supports diagnostic clarity, fosters trust, and operationalises the principles of trustworthy, human-centric AI in real industrial environments. Having established this theoretical groundwork, the  Section 3 presents an overview of existing research and contributions by other authors in this area and Section 4 examines key industrial datasets that underpin research in explainable defect detection and condition monitoring, providing the empirical basis for evaluating XAI techniques across diverse manufacturing contexts.

## 3. Related Work: Explainable AI in Manufacturing

XAI has begun to find application across multiple manufacturing domains, driven by the need for transparent decision support in safety-critical and high-cost industrial environments. Systematic reviews of XAI adoption in smart manufacturing show an increasing trend toward practical deployment of explainability techniques, particularly in predictive maintenance, defect detection, and process optimisation [32]. In the context of predictive maintenance, several studies have highlighted the importance of explainable models to support understanding of equipment failure predictions and decisions made by machine learning systems. Dereci et al. discuss existing XAI approaches for predictive maintenance, underscoring the growing interest in model interpretability in this domain [33]. Similarly, position papers on explainable predictive maintenance identify gaps in how tailored explanations are provided for different user groups in industrial tasks, suggesting that domain-specific XAI design is still under development [34].

Beyond predictive maintenance, XAI methodologies have also been applied to quality control and defect analysis in manufacturing. Marín Díaz proposes integrating clustering with XAI techniques such as SHAP and LIME to support both global and local interpretability of defect prediction models in industrial settings, revealing influential production parameters [35]. The literature also includes conceptual frameworks that emphasise transparency, root-cause analysis, and human interpretability for manufacturing decision support [36]. Despite these advances, existing work typically remains (modality-specific or task-specific), often focusing on a single manufacturing application such as predictive maintenance or quality inspection. There is limited research on (cross-modal XAI generalisation), and few studies treat explainability as an integral part of unified human-in-the-loop industrial workflows. Addressing these gaps motivates the cross-modal, explainability-centred framework proposed in this paper. To highlight how existing XAI research in manufacturing compares to the proposed explainability framework, Table 2 summarises representative prior studies across key capabilities such as predictive maintenance, quality analysis, human-centred interpretability, and multi-modal evaluation.

## 4. Literature Review on Industrial Defect Detection Datasets

The integration of AI into manufacturing has been accelerated by the availability of benchmark datasets that replicate real-world inspection and monitoring tasks. Such datasets shown in Table 3 provide standardised testbeds for developing and validating machine learning and deep learning models, fostering reproducibility and comparability across approaches. Among the most widely utilised resources are the Casting Product Dataset, the GC10-DET dataset, and the MIMII dataset. Collectively, these datasets span multiple modalities—visual inspection through binary classification, defect localisation with multi-class detection, and machine condition monitoring using acoustic data.

### 4.1. Casting Product Dataset

**Figure 4:**
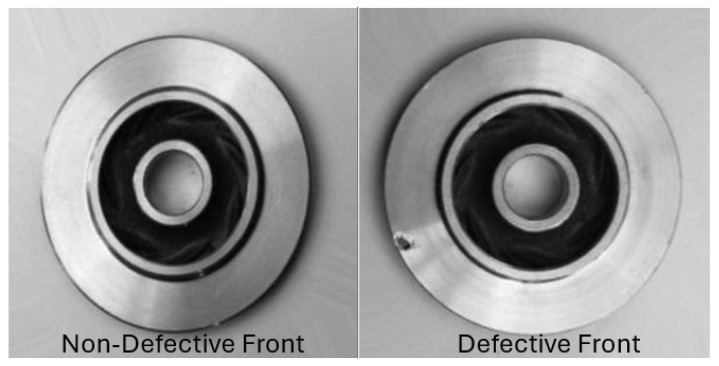
Representative samples from the benchmark datasets used in this study: casting defect image for visual inspection.

The Casting Product Dataset [17] contains 7348 grayscale images of submersible pump impeller castings as shown in Figure 4, categorized as either defective or defect-free. The images (300 × 300 pixels) include instances of shrinkage, blowholes, pinholes, and burrs. This dataset has been extensively used for benchmarking convolutional neural networks (CNNs) and related classification algorithms in automated visual inspection.

From a trustworthiness perspective, this dataset supports research into robustness and accuracy validation of defect detection models. Since binary classification decisions directly affect product acceptance or rejection, ensuring that AI models are interpretable and reliable is essential for gaining operator trust in industrial settings.

### 4.2. GC10-DET: Defects Class and Location Dataset

**Figure 5:**
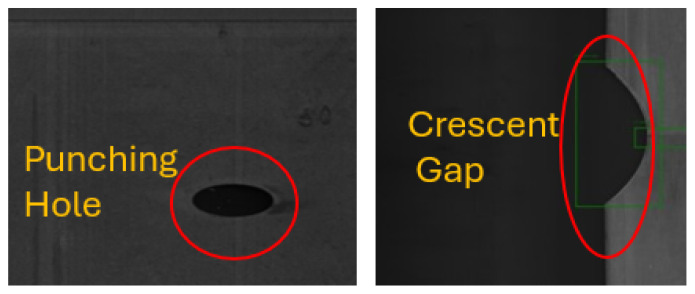
Representative samples from the benchmark datasets used in this study: metal surface defect image from the GC10-DET dataset.

The GC10-DET dataset [18] significantly extends the capabilities of automated visual inspection by offering a comprehensive collection of 2300 steel surface images that encompass 2280 annotated defect instances stored in .xml format. Each annotation specifies a bounding box, allowing the dataset to support both defect classification and localisation tasks. It comprises ten distinct categories of surface imperfections, a few of which are shown in Figure 5: punching (Pu), weld line (Wl), crescent gap (Cg), water spot (Ws), oil spot (Os), silk spot (Ss), inclusion (In), rolled pit (Rp), crease (Cr), and waist folding (Wf). All defects were captured directly from steel sheet surfaces, reflecting realistic industrial inspection conditions.

This dataset provides a platform for testing advanced object detection algorithms such as Faster R-CNN, SSD, and YOLO. Importantly, its spatial annotations facilitate research in XAI, as bounding boxes inherently provide a level of interpretability by visually aligning algorithmic predictions with actual defect regions. In industrial practice, this visual transparency strengthens trust between operators and AI systems, as inspectors can verify the reasoning behind automated decisions.

### 4.3. MIMII Dataset

**Figure 6:**
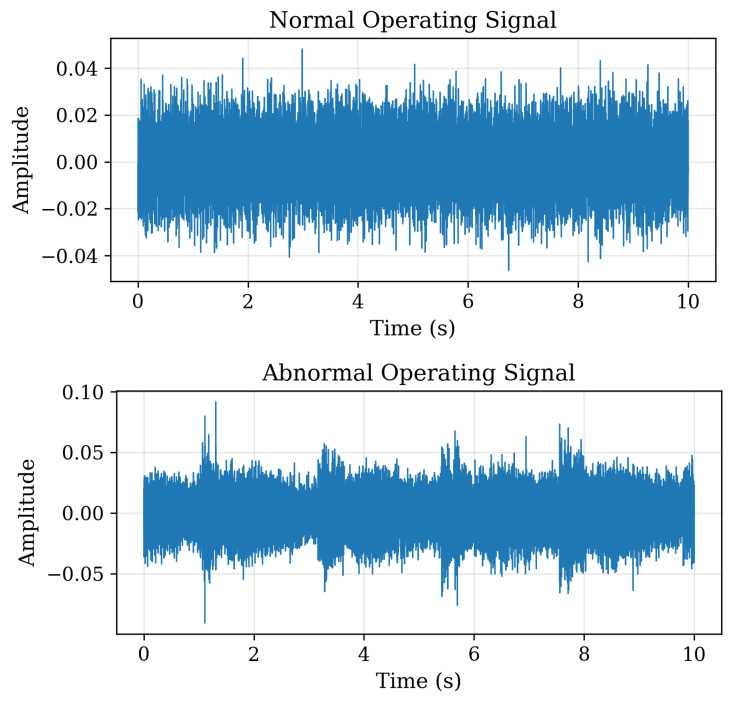
Time-domain representations of acoustic signals obtained from the MIMII pump dataset (ID 06), illustrating normal and abnormal operating conditions. The abnormal signal exhibits increased amplitude variations compared to normal operation.

The MIMII dataset (Malfunctioning Industrial Machine Investigation and Inspection) [19] introduces an acoustic monitoring dimension, with sound recordings from fans, pumps, valves, and slide rails. Each recording includes both normal and abnormal operating conditions as shown in Figure 6, collected in realistic factory environments with background noise.

This dataset underpins research into acoustic anomaly detection for predictive maintenance. It has been widely employed in studies utilising spectrogram-based CNNs, autoencoders, and unsupervised anomaly detection. For AI trustworthiness, MIMII enables investigation into uncertainty estimation and anomaly explanation, where sound patterns can be correlated with mechanical faults. By connecting audible signatures to equipment health, the dataset supports transparent and justifiable decision-making in machine condition monitoring.

### 4.4. Summary and Link to AI Trustworthiness

Taken together, these datasets represent complementary aspects of industrial monitoring: binary defect detection, multi-class defect localisation, and acoustic anomaly identification. Beyond enabling algorithmic development, they provide avenues for exploring key trustworthiness dimensions of AI across diverse manufacturing contexts.

For instance, vision-based datasets such as the Casting Product and Defects Class and Location datasets enable the study of explainability in automated inspection systems, where AI models are deployed to identify surface flaws, verify geometric integrity, and detect micro-defects during high-throughput production. These systems mirror real-world inspection pipelines used in casting, forging, and additive manufacturing, where explainable AI can justify defect classifications to operators and improve rework accuracy.

Meanwhile, acoustic datasets like MIMII simulate continuous equipment monitoring environments, where sound and vibration data are captured from motors, compressors, and pumps to diagnose early-stage mechanical faults. Such datasets are particularly relevant for predictive maintenance and condition-based monitoring, where explainable models not only flag anomalies but also indicate which spectral or temporal features, such as variations in frequency bands or energy levels, contributed to the detection outcome.

Extending beyond visual and acoustic sensing, the same principles of explainability apply to multivariate time-series data obtained from process sensors measuring parameters such as temperature, pressure, and flow rate. In these contexts, XAI can help identify which process variables exert the greatest influence on deviations in quality or energy efficiency, facilitating real-time, closed-loop decision-making in smart manufacturing systems.

Collectively, these datasets and modalities encompass the core operational pillars of modern manufacturing—product inspection, machine health monitoring, and process optimisation. Product inspection focuses on the early detection and classification of surface defects, dimensional deviations, or assembly errors to ensure consistent quality during production. Machine health monitoring involves continuous tracking of acoustic and vibration signatures to identify wear, imbalance, or component degradation before critical failures occur, thereby improving equipment reliability and reducing unplanned downtime. Process optimisation extends these principles to multivariate sensor data, enabling real-time adjustment of process parameters such as temperature, pressure, or feed rate to enhance productivity, reduce energy consumption, and maintain stable operational performance. By integrating explainability across these domains, AI-driven systems can bridge human interpretability with computational intelligence, ensuring that automated decisions remain transparent, accountable, and actionable within industrial environments.

By grounding trustworthy AI research in these publicly available datasets, the field moves closer to developing industrial systems that are not only accurate but also interpretable, verifiable, and aligned with human decision-making processes. Such integration ensures that AI systems deployed on factory floors and production lines can operate transparently under real-world conditions providing engineers with meaningful insights into why specific predictions or classifications are made. This alignment between algorithmic intelligence and operator understanding is essential for fostering confidence in automation and ensuring traceability in decision pathways.

Furthermore, establishing explainable methodologies across domains such as aerospace, automotive, and high-value manufacturing is a critical enabler for the adoption of AI in safety-critical and quality-sensitive applications. In these sectors, where even minor misclassifications can lead to significant operational or economic consequences, XAI provides the foundation for auditing model behavior, verifying compliance with industrial standards, and ensuring that AI-driven recommendations remain consistent with domain expertise and regulatory expectations. Building on these principles, the next section delves into how XAI helps make AI-driven manufacturing systems more transparent and understandable, highlighting its impact across visual inspection, acoustic monitoring, and sensor-based time-series analysis. The insights gained from this review set the stage for the following Section 5, which explores how XAI techniques contribute to transparency and diagnostic understanding across the different manufacturing modalities represented by these datasets.

## 5. The Role of XAI in Manufacturing

Building upon the previously discussed datasets and their industrial relevance, this section focuses on how XAI contributes to transparency within manufacturing systems. The increasing reliance on AI-driven models for inspection, monitoring, and maintenance tasks demands that their decisions be interpretable and aligned with physical process behaviour. However, the opacity of complex deep learning architectures often limits user confidence and hinders accountability in high-stake environments. XAI addresses this limitation by offering methods that clarify the reasoning behind AI outputs. In manufacturing, such transparency is vital: engineers must be able to validate that a model’s decision is grounded in relevant defect indicators rather than spurious patterns.

In this section, we examine how XAI contributes to transparency in manufacturing by considering two critical data modalities: (i) vision-based inspection systems for surface defect detection and (ii) time-series sensor data analysed with unsupervised models for acoustic anomaly detection. Together, these perspectives provide a comprehensive view of how XAI enhances interpretability across heterogeneous manufacturing tasks.

### 5.1. XAI for Vision-Based Models

Computer vision has become a cornerstone of automated manufacturing, where the accurate detection of small surface defects can prevent costly quality failures. Deep learning-based detectors such as convolutional neural networks and region-based architectures have set benchmarks in defect recognition tasks [1,2,3]. Despite their accuracy, these models function as black boxes, producing classifications or bounding boxes without exposing the rationale behind their outputs. This opacity is problematic in industrial contexts where quality engineers require clear evidence that the model has focused on physically relevant features of the product.

To mitigate this issue, XAI methods designed for vision tasks have been widely adopted. Techniques such as Gradient-weighted Class Activation Mapping (Grad-CAM), saliency maps, occlusion analysis, and Integrated Gradients generate visual heatmaps that highlight the image regions most responsible for a prediction. These approaches translate the internal activations of deep neural networks into interpretable visual cues that help engineers validate whether the network has correctly focused on defect-relevant regions rather than background noise or irrelevant features [37,38].

#### 5.1.1. Saliency Maps

Saliency maps compute the gradient of the model output with respect to each input pixel, capturing how small perturbations in pixel intensity affect the final class score [39]. For a model f(x) and class score $S_{c}\left(x\right)$, the saliency at pixel $x_{i}$ is expressed as:

(1) $$M_{i}=\left|\frac{\partial S_{c}\left(x\right)}{\partial x_{i}}\right|$$

This method highlights the most sensitive pixels driving the classification decision, offering a first-order approximation of the model’s local behaviour. In manufacturing defect detection, high-intensity regions in a saliency map typically correspond to cracks, pores, or irregular textures that influenced the network’s output.

#### 5.1.2. Gradient-Weighted Class Activation Mapping

Grad-CAM [40] extends this concept by leveraging the gradient information flowing into the last convolutional layer of a CNN. For class *c*, the importance of feature map $A^{k}$ is determined as follows:

(2) $$\alpha_{k}^{c}=\frac{1}{Z}\sum_{i}\sum_{j}\frac{\partial y^{c}}{\partial A_{ij}^{k}}$$

where *Z* is the number of spatial locations. The Grad-CAM heatmap is then obtained as follows:

(3) $$L_{\mathrm{Grad}-\mathrm{CAM}}^{c}=\mathrm{ReLU}\left(\sum_{k}\alpha_{k}^{c}A^{k}\right)$$

This produces a coarse localisation map showing which image regions contribute most to a specific class prediction. In defect inspection, Grad-CAM helps visualise whether the model attends to actual defect areas rather than uniform surfaces or shadows.

#### 5.1.3. Integrated Gradients

Integrated Gradients (IG) [41] attributes importance to each input feature by integrating gradients along a straight path from a baseline (e.g., a black image) $x^{\prime }$ to the actual input x. For feature *i*, the attribution is

(4) $${\mathrm{IG}}_{i}\left(x\right)=\left(x_{i}-x_{i}^{\prime }\right)\int_{\alpha =0}^{1}\frac{\partial F(x^{\prime }+\alpha \left(x-x^{\prime }\right))}{\partial x_{i}}\;d\alpha$$

Unlike raw gradients, IG mitigates the problem of gradient saturation and yields smoother, more stable attributions. In industrial contexts, it provides a cumulative importance estimate, indicating which structural or texture features most influence the defect classification outcome.

#### 5.1.4. Occlusion Sensitivity

Occlusion analysis, introduced by Zeiler and Fergus [42], measures how the class score changes when local regions of an image are masked or replaced. For class *c*, the importance of region *R* is given by

(5) $$\Delta S_{c}\left(R\right)=S_{c}\left(x\right)-S_{c}\left(x\setminus R\right)$$

where x∖R denotes the input with region *R* occluded. This technique identifies the most critical areas for classification by directly testing the model’s sensitivity to missing visual information—an effective strategy for verifying the robustness of defect localisation models in quality inspection.

Together, these visualisation methods bridge deep network reasoning with human interpretation, allowing practitioners to confirm whether model attention aligns with ground-truth defect locations. Their complementary nature—gradient-based (Saliency, Grad-CAM, IG) versus perturbation-based (Occlusion) provides a comprehensive toolkit for interpreting convolutional models used in manufacturing inspection [37,38].

In our study, we applied these techniques to two datasets. Using the casting product dataset [17], YOLOv8n was trained for defect detection, and its outputs were interpreted through Grad-CAM, Integrated Gradients, saliency maps, and occlusion. These explanations revealed how the detector localised casting defects, confirming whether predictions aligned with ground-truth anomalies. In parallel, the Defects Class and Location dataset [18] was used to train Faster R-CNN for defect classification and localisation. Explanations from Grad-CAM and saliency mapping were employed to analyse bounding box predictions, providing insight into how the network distinguished between defect categories and their spatial placement. This combination of models and interpretability methods demonstrates that XAI strengthens the reliability of computer vision systems in manufacturing by coupling detection accuracy with transparent reasoning.

### 5.2. XAI for Time-Series and Unsupervised Models

While visual inspection addresses visible surface anomalies, many industrial systems rely on continuous sensor monitoring, producing time-series data such as sound or vibration signals. These modalities are essential for tasks such as predictive maintenance and fault diagnosis. A challenge in this setting is that labeled datasets are often scarce, making unsupervised learning approaches more practical. Methods like Isolation Forests or autoencoders detect deviations from normal operating behavior but, as unlabeled models, their decision-making is even less transparent than that of supervised vision models [43].

Recent research has extended XAI to such contexts, showing that techniques like SHAP—originally developed for tabular and image data can be adapted to univariate and multivariate time-series [12,37,44]. Other studies have proposed KernelSHAP-based explanations for unsupervised models, generating local instance-level interpretability [45], while some frameworks also provide global insights into anomaly detection mechanisms [46,47]. Collectively, these advances illustrate that interpretability can be achieved even in the absence of labeled data, enhancing both trust and diagnostic capability.

SHAP attributes the contribution of each feature by applying Shapley values from cooperative game theory [44]. For a model *f* with feature set *F*, the Shapley value $\phi_{i}$ of feature *i* is defined as:

(6) $$\phi_{i}=\sum_{S\subseteq F\setminus \{i\}}\frac{|S|!(|F|-|S|-1)!}{|F|!}[f(S\cup \{i\})-f(S)]$$

This ensures a fair distribution of feature contributions by averaging over all possible subsets *S* of features. SHAP provides both local interpretability (per-instance explanations) and global interpretability (average importance across the dataset), enabling transparent understanding of model behaviour in sensor-based fault detection tasks.

Our contribution builds on this direction using the MIMII dataset [19], which contains acoustic recordings of industrial machines. We trained an Isolation Forest exclusively on normal signals to learn the baseline of machine operation and applied it for anomaly detection. SHAP was then integrated to identify which acoustic features—including root mean square (RMS) energy, zero-crossing rate (ZCR), and Mel-frequency cepstral coefficients (MFCCs), drove the classification of abnormal segments. This enabled fine-grained interpretability, linking anomaly decisions to specific signal properties and offering actionable insights into machine behavior. In this way, XAI transformed a purely statistical detector into a transparent tool for acoustic monitoring in manufacturing.

### 5.3. Bridging Modalities

Existing literature on XAI in manufacturing has largely concentrated on a single modality, either focusing on vision-based defect detection or on sensor-driven anomaly analysis. By contrast, our study integrates XAI across both domains. We applied Grad-CAM, saliency, Integrated Gradients, and occlusion maps to deep learning models for defect detection, and complemented this with SHAP applied to time-series data modeled by Isolation Forests. This cross-modal perspective demonstrates that explainability can be systematically embedded into diverse manufacturing pipelines, from supervised vision models to unsupervised acoustic detectors.

By unifying these approaches, our work advances the goal of trustworthy AI in manufacturing, showing that interpretability is not restricted to one data type or model family but can be consistently applied across heterogeneous industrial tasks. This establishes a foundation for the broader adoption of XAI in production systems where both accuracy and transparency are paramount. After examining the theoretical and practical role of XAI in enhancing transparency, Section 6 presents the proposed methodology, describing how explainability is systematically integrated into AI pipelines for visual and acoustic inspection tasks.

## 6. Methodology

The proposed methodology is designed as a unified framework for integrating XAI into manufacturing applications, with the primary objective of investigating how XAI techniques can be used to open the black-box behaviour of AI systems commonly encountered in industrial practice. Rather than optimising or comparing machine learning models, the study treats model selection as a secondary consideration and employs the chosen models as representative black-box decision-makers, enabling focused evaluation of explainability. The framework follows a common sequence of steps, data acquisition and preprocessing, model training, application of explainability techniques, and evaluation—adapted to different manufacturing data modalities. To demonstrate the breadth and generalisability of the approach, the methodology is applied to three representative case studies: image classification of casting defects, object detection of metal surface defects, and acoustic anomaly detection in machine sound.

To support the explainability-centred objective, the study deliberately employs diverse model architectures and learning paradigms, including supervised convolutional neural networks for visual inspection, region-based object detectors for defect localisation, and unsupervised anomaly detection models for acoustic condition monitoring. This diversity is intentional and enables systematic assessment of whether XAI methods can provide meaningful and physically interpretable explanations independent of model structure, task formulation, or supervision strategy. In the visual inspection domain, YOLOv8n is used as a lightweight representative of convolutional models deployed in real-time industrial inspection systems. The intent is not to demonstrate superiority over alternative CNN architectures, but to evaluate whether gradient- and activation-based XAI techniques can successfully expose the internal reasoning of a commonly adopted deep learning classifier in quality control scenarios. For defect localisation, Faster R-CNN is employed as a representative region-based detector widely used in industrial and research contexts. Its two-stage architecture provides a suitable testbed for examining whether explainability techniques can align model attention with physically meaningful defect regions rather than background artefacts. In the acoustic condition monitoring task, an Isolation Forest is selected to represent unsupervised anomaly detection, reflecting the practical reality that labelled fault data are often unavailable in manufacturing environments. Here, the emphasis is not on anomaly detection performance in isolation, but on assessing whether feature-level explainability methods such as SHAP can transform an inherently opaque statistical model into a transparent and interpretable diagnostic tool.

By intentionally avoiding model comparison and hyperparameter optimisation, the study maintains a clear focus on explainability as the central experimental variable. The use of heterogeneous models allows the work to demonstrate that XAI techniques can be systematically applied across diverse industrial AI pipelines to support transparency, trust, and human-in-the-loop decision-making. Each case study is described in detail below.

### 6.1. Casting Defects: Image Classification with YOLOv8 and Explainability

**Figure 7:**
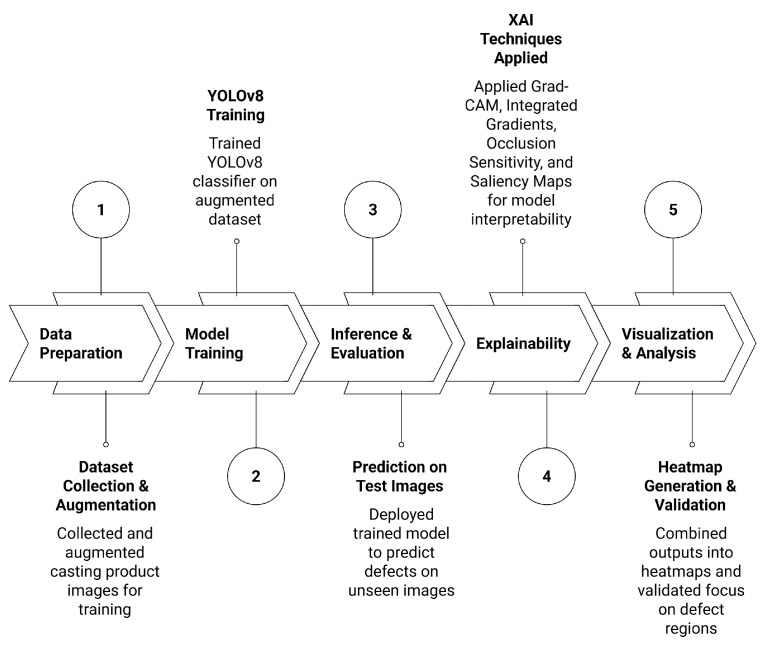
Workflow of the visual inspection experiment using YOLOv8 and explainable AI (XAI) techniques. The process includes: (1) data preparation through image collection and augmentation, (2) model training with YOLOv8, (3) inference and evaluation on test images, (4) application of Grad-CAM, Integrated Gradients, Occlusion, and Saliency methods for explainability, and (5) visualisation and validation of heatmaps to confirm focus on true defect regions.

The Casting Defects dataset [17] was employed to investigate the role of explainable XAI in automated defect detection. This dataset contains grayscale images of automotive casting components divided into two categories: ok_front, representing defect-free samples, and def_front, representing defective castings with surface irregularities such as blowholes and cracks. Images are organised into train and test directories with subfolders corresponding to each class. Although the dataset is grayscale, images were processed as three-channel tensors to meet the input requirements of the chosen model. The complete workflow for this experiment, from data preparation to explainability and validation, is illustrated in Figure 7.

To increase dataset diversity and reduce orientation bias, in-place rotation augmentation was applied to both the training and testing sets. Each sample was rotated by $45^{{}^{\circ}}$, $90^{{}^{\circ}}$, and $135^{{}^{\circ}}$ using affine transformations implemented in OpenCV. A reflective padding mode was employed to avoid artificial edges caused by rotation. Augmented images were stored alongside the originals, effectively expanding the dataset size fourfold. This preprocessing strategy improved model generalisation and robustness against positional variation.

For classification, the YOLOv8n classifier (yolov8n-cls.pt) was adopted. This lightweight convolutional neural network comprises a convolutional backbone and a linear classification head. Fine-tuning was carried out using the Ultralytics API with default settings for classification tasks. Training employed the categorical cross-entropy loss, defined as

(7) $$L=-\sum_{c=1}^{C}y_{c}\log{\hat{y}}_{c},$$

where $y_{c}$ is the ground-truth label and ${\hat{y}}_{c}$ is the predicted probability for class *c*. Since the task is binary, C=2. The network was trained for three epochs with a batch size of forty and an input resolution of 300×300 pixels. Training was performed on GPU hardware when available, and on CPU otherwise. The best-performing checkpoint was stored at runs/classify/train3/weights/best.pt and was subsequently used for all explainability experiments. The overall pipeline followed in this study is summarized in Algorithm 1.
**Algorithm 1** Casting Defect Classification and Explainability Framework1:**Input:** Dataset D with classes *ok_front*, *def_front*2:**Output:** Trained YOLOv8n classifier with explanations3:Load dataset, convert grayscale to 3-channel tensors4:Apply rotation augmentation ($45^{{}^{\circ}}$, $90^{{}^{\circ}}$, $135^{{}^{\circ}}$)5:Train YOLOv8n classifier with cross-entropy loss6:Evaluate and save best checkpoint7:For each test sample, compute explanations:
Saliency MapsGrad-CAM (SmoothGradCAM++)Integrated GradientsOcclusion Sensitivity8:Normalise and overlay explanation maps on original images9:Visualise and interpret model focus on defect regions

These training parameters follow standard fine-tuning practices for YOLO-based models reported in the literature and official Ultralytics YOLOv8 implementations, where pretrained weights and limited training epochs are commonly used for industrial inspection tasks to ensure stable convergence on moderate-sized datasets [48,49].

To provide insights into the decision-making process of the YOLOv8n classifier, four complementary XAI techniques were employed. Saliency maps were computed by evaluating the gradient of the class score with respect to each input pixel, which measures how sensitive the model’s prediction is to changes in individual pixel intensities. In simple terms, pixels with larger gradient values have a greater influence on the final decision, highlighting the specific regions in the image that most strongly affect the classification outcome.

(8) $$M_{i}=\left|\frac{\partial S_{c}\left(x\right)}{\partial x_{i}}\right|$$

where $S_{c}\left(x\right)$ denotes the logit for class *c*. Gradient-weighted Class Activation Mapping (Grad-CAM) was also used to generate coarse, class-discriminative heatmaps from the final convolutional layer. Feature map weights were calculated as

(9) $$\alpha_{k}^{c}=\frac{1}{Z}\sum_{i}\sum_{j}\frac{\partial y^{c}}{\partial A_{ij}^{k}},\;L_{\mathrm{Grad}-\mathrm{CAM}}^{c}=\mathrm{ReLU}\left(\sum_{k}\alpha_{k}^{c}A^{k}\right),$$

where $A^{k}$ are the feature maps and *Z* is the normalisation factor. A more stable variant, SmoothGradCAM++, was employed to improve localisation around fine-grained defect cues.

Integrated Gradients were applied to capture attributions along a continuous path from a baseline input $x^{\prime }$ to the actual input x. For feature *i*, the attribution is defined as

(10) $${\mathrm{IG}}_{i}\left(x\right)=\left(x_{i}-x_{i}^{\prime }\right)\int_{0}^{1}\frac{\partial F(x^{\prime }+\alpha \left(x-x^{\prime }\right))}{\partial x_{i}}d\alpha .$$

This technique provides smoother and more reliable attributions than raw gradients by reducing sensitivity to gradient saturation.

Finally, Occlusion Sensitivity was used as a perturbation-based method to evaluate the importance of local regions. In this approach, an image region *R* is systematically masked, and the change in the class score is recorded:

(11) $$\Delta S_{c}\left(R\right)=S_{c}\left(x\right)-S_{c}\left(x\setminus R\right),$$

where x∖R denotes the occluded image. Scanning the mask across the input produced a spatial importance map, which highlighted defect-sensitive regions in the castings.

For each explainability method, the resulting heatmaps were scaled between 0 and 1 and visually overlaid on the original casting images using consistent color maps. This allowed an intuitive inspection of where the model was “looking” when making its decisions. In other words, the highlighted areas show which parts of the image most influenced the classification. Combining gradient-based, activation-based, and perturbation-based methods gave a complete picture of the model’s reasoning and helped confirm that it focused on actual defect regions rather than irrelevant background patterns.

### 6.2. Metal Surface Defects: Object Detection with Faster R-CNN and Explainability

The Defects Class and Location dataset [18] was employed to evaluate explainability in the context of object detection for metal surface inspection. This dataset comprises images of metal surfaces annotated with bounding boxes in Pascal VOC XML format, describing the location and type of surface defects. Each image is associated with one or more defect regions, and the labels correspond to defect classes obtained from the folder structure. The complete workflow followed in this study is summarised in Algorithm 2.
**Algorithm 2** Metal Surface Defect Detection and Explainability Framework  1:**Input:** Dataset D of annotated metal surface images  2:**Output:** Trained Faster R-CNN model with explanations  3:Parse Pascal VOC XML files to extract bounding boxes and defect classes  4:Normalise bounding box coordinates by image dimensions  5:Split dataset into training (80%) and validation (20%) subsets  6:Apply oversampling to balance under-represented defect classes  7:Construct a PyTorch dataset returning (x,y) pairs with images and targets  8:Initialise Faster R-CNN with ResNet-50 FPN backbone and replace detection head to match number of classes  9:Train model for 50 epochs with batch size 4 using SGD optimiser (lr=0.005, momentum =0.9, weight decay $=5\times 10^{-4}$)10:Optimise multi-task loss $L=L_{cls}+\lambda L_{box}$11:Save the best-performing model checkpoint12:For each test image, compute visual explanations:
Saliency Maps: compute gradients of class logits w.r.t. input pixelsGrad-CAM: generate class-discriminative heatmaps from convolutional layers in the detection backbone
13:Normalise explanation maps to [0,1] and overlay on input images14:Compare predicted bounding boxes with ground-truth annotations15:Assess whether explanation highlights coincide with true defect regions

To construct the dataset, XML files were parsed to extract bounding box coordinates and class labels. Each bounding box was normalised with respect to the image dimensions to ensure numerical stability during training. The dataset was divided into training and validation subsets using an 80/20 split, stratified by defect class. To address class imbalance, oversampling of under-represented classes was performed until each class reached parity with the largest class. A custom PyTorch (version 2.5.1, CUDA 12.1) Dataset was implemented to return paired samples (x,y), where *x* is the input image and y=boxes,labels represents the ground-truth bounding boxes and class identifiers.

The availability of ground-truth bounding box annotations in the GC10-DET dataset enables quantitative validation of defect localisation using standard object detection metrics. In this study, predicted bounding boxes produced by the Faster R-CNN model are quantitatively evaluated against ground-truth annotations, achieving peak localisation performance of approximately mIoU=0.44 and mAP@0.5=0.61, which provides sufficient confidence in the plausibility of the localisation results. Explainability analysis is then applied to these validated predictions, and the quality of XAI is assessed by examining the spatial alignment between high-activation regions in the explanation maps and the annotated defect locations. Importantly, localisation accuracy is not treated as the primary optimisation objective; rather, it serves as contextual grounding to ensure that explainability results are interpreted in relation to reliable defect detection rather than evaluated in isolation.

The detection model was based on the Faster R-CNN architecture, implemented in the TorchVision library. A ResNet-50 backbone with Feature Pyramid Network (FPN) was adopted, initialised with weights pretrained on COCO. The classification head was modified to accommodate the number of defect classes in the dataset plus the background class. Training was conducted for fifty epochs with a batch size of four. The stochastic gradient descent (SGD) optimiser was used with a learning rate of 0.005, momentum of 0.9, and weight decay of $5\times 10^{-4}$. This training configuration follows widely adopted practices in Faster R-CNN–based industrial defect detection, as established in the original Faster R-CNN formulation and subsequent object detection literature, providing stable convergence and reliable localisation performance without task-specific hyperparameter tuning, which aligns with the explainability-centred objective of this study rather than performance optimisation [50]. The model was trained to minimise the multi-task loss function of Faster R-CNN, which combines a classification loss $L_{cls}$ and a bounding-box regression loss $L_{box}$:

(12) $$L=L_{cls}+\lambda L_{box},$$

where λ balances the contribution of the regression term.

The explainability analysis was carried out using both gradient-based and activation-based attribution methods. Saliency maps were obtained by computing the absolute gradient of the detection score with respect to input pixels, thereby identifying sensitive regions. Grad-CAM was employed on the backbone convolutional layers to generate class-discriminative activation maps, defined as

(13) $$L_{\mathrm{Grad}-\mathrm{CAM}}^{c}=\mathrm{ReLU}\left(\sum_{k}\alpha_{k}^{c}A^{k}\right),$$

where $A^{k}$ denotes the feature maps and $\alpha_{k}^{c}$ are weights derived from the gradients of the class score with respect to $A^{k}$. These explanation maps were normalised and overlaid on the corresponding input images to provide interpretable insights into the model’s decision-making process.

### 6.3. Acoustic Anomaly Detection: Isolation Forest with XAI for Time-Series Data

**Figure 8:**
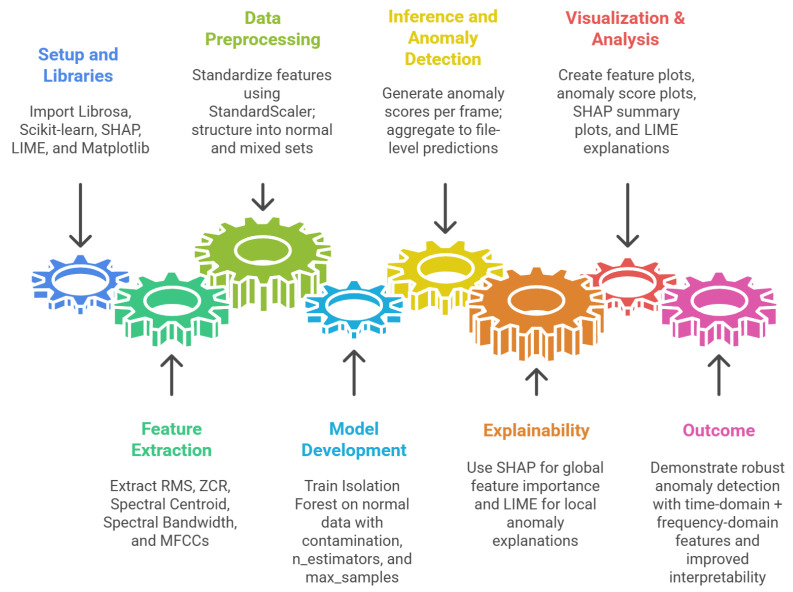
Workflow of the acoustic anomaly detection and explainability experiment. The process begins with setup and library configuration, followed by feature extraction of acoustic descriptors such as RMS, zero-crossing rate (ZCR), spectral centroid, spectral bandwidth, and MFCCs. After feature standardisation and dataset structuring, an Isolation Forest model is trained on normal data to detect anomalies. SHAP is then applied for global feature importance analysis, and the results are visualised through anomaly score plots and SHAP summary plots to interpret model behavior and validate feature contributions.

In this part of the study, we investigated anomaly detection in acoustic signals using an unsupervised learning approach combined with explainable artificial intelligence (XAI). The dataset consisted of audio recordings of machines under both normal and abnormal operating conditions, stored as .wav files in separate normal/ and abnormal/ directories. Each recording was sampled at 16 kHz, preserving both low- and high-frequency characteristics. The complete workflow for acoustic anomaly detection and explainability is detailed in Algorithm 3 and illustrated in Figure 8.
**Algorithm 3** Acoustic Anomaly Detection and Explainability Framework   **Input:** Audio dataset D with normal/ and abnormal/ recordings   **Output:** Trained Isolation Forest with SHAP explanations  3:Load all audio files and resample to 16 kHzSegment each recording into frames of length 1024 with hop size 512Extract per-frame features: RMS, ZCR, Centroid, Bandwidth, MFCC1–MFCC3  6:Normalise all features using z-score standardisationTrain Isolation Forest on normal samples with contamination parameter 0.05For each test recording:
Compute anomaly scores $s(f_{t})$ for each frameApply threshold θ (90th percentile) to detect anomaliesMap frame indices back to time domain and highlight anomalous segments  9:Apply SHAP to obtain local and global feature attributionsAggregate predictions at the file level (abnormal if >30% anomalous frames)Compute confusion matrix, precision, recall, and F1-score12:Visualise waveform plots with shaded anomalies and overlay XAI explanations

Feature Extraction Each audio signal was divided into overlapping frames of length 1024 samples with a hop size of 512. From every frame, seven features were extracted using the Librosa library: Root Mean Square (RMS) energy, Zero-Crossing Rate (ZCR), Spectral Centroid, Spectral Bandwidth, and the first three Mel-Frequency Cepstral Coefficients (MFCCs). This resulted in a feature vector of dimension d=7 for each frame *t*:

(14) $$f_{t}=\left[{\mathrm{RMS}}_{t},{\mathrm{ZCR}}_{t},{\mathrm{Centroid}}_{t},{\mathrm{Bandwidth}}_{t},{\mathrm{MFCC}1}_{t},{\mathrm{MFCC}2}_{t},{\mathrm{MFCC}3}_{t}\right].$$

The selection of acoustic features in this study is intentionally guided by human interpretability rather than by maximising model performance. The chosen indicators RMS energy, zero-crossing rate, spectral centroid, spectral bandwidth, and low-order MFCCs are widely used in acoustic condition monitoring because they correspond to perceptually meaningful properties of sound, such as loudness, roughness, brightness, and timbral variation. These characteristics are directly related to what human operators perceive as abnormal machine behaviour, including increased vibration, harsh impacts, tonal shifts, or broadband noise.

From a human-in-the-loop perspective, this feature design supports explainability by ensuring that XAI outputs can be related back to operators’ practical auditory skills rather than abstract latent representations. For example, elevated RMS energy corresponds to louder or more energetic sounds, while changes in spectral centroid and MFCCs reflect shifts in tonal balance that are often audible during mechanical degradation. This approach aligns with prior work on human-centred and explainable acoustic monitoring, where interpretable features are preferred to preserve operator understanding and reduce over-reliance on opaque AI systems. To ensure comparability across features, z-score normalisation was applied:

(15) $$f_{t,i}^{\prime }=\frac{f_{t,i}-\mu_{i}}{\sigma_{i}},$$

where $\mu_{i}$ and $\sigma_{i}$ are the mean and standard deviation of feature *i* computed from the training set.

Anomaly Detection Model An Isolation Forest (IF) was trained exclusively on normal feature vectors to learn the statistical profile of healthy machine sounds. The contamination parameter was set to 0.05 to reflect the expected proportion of anomalies. For each frame $f_{t}$, the anomaly score was computed as the negative decision function:

(16) $$s\left(f_{t}\right)=-h\left(f_{t}\right),$$

where $h(f_{t})$ denotes the average path length of $f_{t}$ across the isolation trees. Frames with high anomaly scores were more likely to correspond to faulty machine states.

Temporal Anomaly Mapping Predictions were generated frame by frame, and the anomaly scores were mapped back to the time domain using

(17) $$t=\frac{n\cdot H}{sr},$$

where *n* is the frame index, H=512 is the hop size, and sr = 16,000 Hz is the sampling rate. A threshold θ corresponding to the 90th percentile of anomaly scores was chosen:

(18) $$\theta ={\mathrm{Percentile}}_{90}\left(s\left(f_{1}\right),\ldots ,s\left(f_{T}\right)\right),$$

and frames with $s(f_{t})>\theta$ were flagged as anomalies. These anomalous regions were highlighted on the waveform plots, enabling visual inspection of fault events over time.

Explainability To enhance transparency, the SHAP (Shapley Additive Explanations) method was applied to interpret the model’s predictions. SHAP provided both local explanations—using waterfall plots for individual anomalies and global explanations using beeswarm plots across the entire dataset. For each time frame *t*, SHAP computed per-feature contribution values $\phi_{i}^{t}$, satisfying the additive property of feature attributions:

(19) $$f\left(f_{t}\right)=\phi_{0}+\sum_{i=1}^{d}\phi_{i}^{t}.$$

Evaluation For file-level detection, a recording was considered abnormal if more than 30% of its frames were flagged as anomalies. Performance was assessed using a confusion matrix and standard metrics: precision, recall, and F1-score. In addition, waveform plots with shaded anomaly regions and XAI visualisations were generated for qualitative assessment.

This methodology combined domain-specific audio feature extraction, unsupervised learning via Isolation Forest, and multi-level explainability using SHAP. It ensured both accurate anomaly detection and interpretability, addressing the requirements of industrial condition monitoring. The following Section 7 presents and analyses the experimental results obtained from the proposed methodology, demonstrating how XAI enhances interpretability and trust in AI-driven quality and condition monitoring systems.

## 7. Results and Discussion

This section presents the explainability outcomes obtained across two complementary sensing regimes in manufacturing: vision-based inspection and acoustic condition monitoring. We first analyse the vision-based pipelines—casting defect classification (YOLOv8n) and metal surface defect detection and localisation (Faster R-CNN)—with a focus on how post-hoc explanations (Grad-CAM, Integrated Gradients, Saliency, and Occlusion) reveal the spatial evidence supporting each decision and align with physically meaningful defect cues. We then examine the acoustic pipeline (Isolation Forest on MIMII-style recordings), where feature-level attributions from SHAP clarify the temporal–spectral characteristics driving anomaly flags. Throughout, our emphasis is on transparency, traceability, and operator interpretability: rather than reporting accuracy as the primary endpoint, we assess whether explanations are consistent with domain knowledge, expose failure modes, and provide auditable justification suitable for deployment in industrial settings.

### 7.1. Vision-Based Explainability Results

This subsection presents explainability results from two vision-based manufacturing pipelines: casting defect classification using YOLOv8n and metal surface defect detection using Faster R-CNN. Before applying explainability techniques, both models were evaluated using standard performance metrics to ensure reliable behaviour (accuracy, precision, recall, and F1-score for YOLOv8n; IoU and mAP for Faster R-CNN). This confirms that the explanations reflect functioning industrial AI systems rather than poor model performance. While all XAI methods highlight regions relevant to the models’ decisions, noticeable discrepancies are observed. Gradient-based methods such as Grad-CAM produce smoother, class-focused heatmaps, whereas saliency-based approaches are more sensitive to local variations and background noise. These differences show that XAI provides complementary, not definitive, explanations, reinforcing the need for cautious interpretation supported by domain knowledge in safety-critical manufacturing contexts.

**Figure 9:**
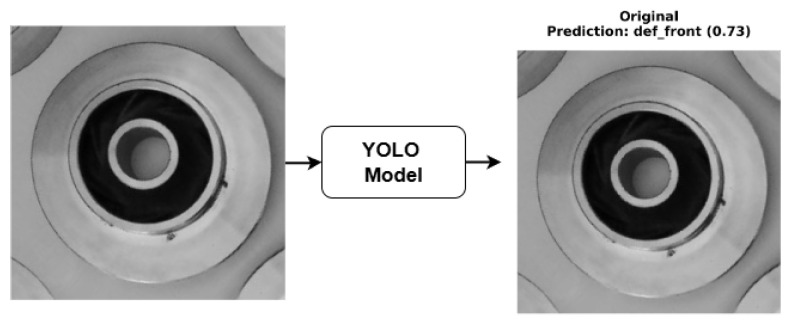
Traditional/black-box AI prediction.

**Figure 10:**
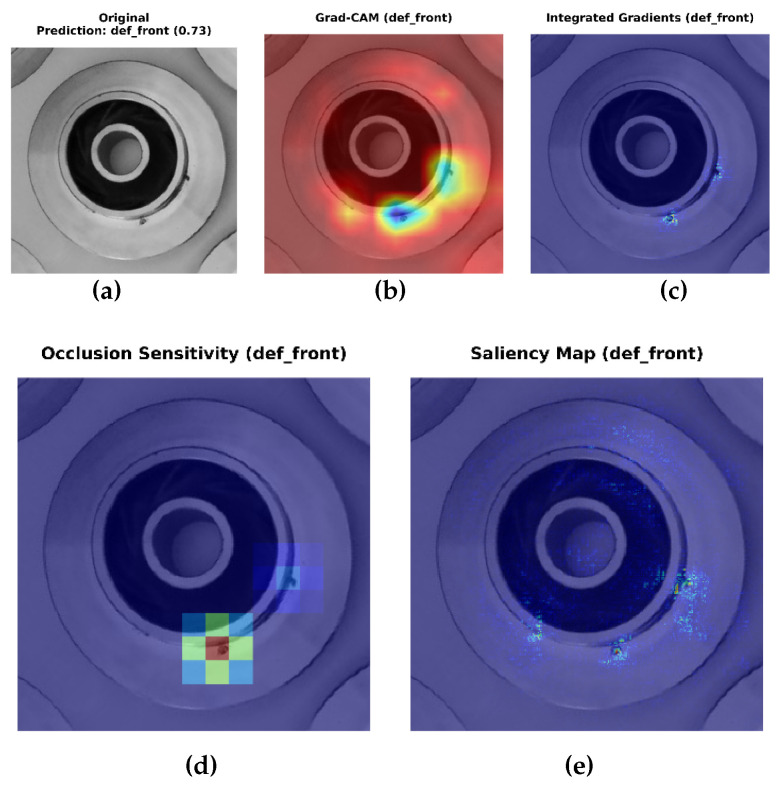
Explainability results for the YOLOv8 classifier on casting defect detection. (**a**) Original input image, (**b**) Grad-CAM heatmap, (**c**) Integrated Gradients attribution, (**d**) Occlusion Sensitivity map, and (**e**) Saliency map.

In conventional computer vision pipelines, the user typically receives only the final model prediction, such as a class label or bounding box without any visibility into the reasoning process behind it. As illustrated in Figure 9, the classifier can indicate whether a casting is “defective” or “non-defective,” but this output alone provides limited diagnostic value to an operator. However, through the integration of XAI techniques, the same decision can be decomposed into interpretable visual evidence (Figure 10). Thus, explainability not only communicates the outcome but also reveals why the model reached it, bridging the gap between algorithmic prediction and human understanding.

For the casting defect classification task, the YOLOv8n classifier was evaluated using four gradient- and perturbation-based explanation methods: Grad-CAM, Integrated Gradients, Saliency Maps, and Occlusion Sensitivity. Grad-CAM visualisations (Figure 10) consistently highlighted localised regions corresponding to surface imperfections such as blowholes, cracks, and uneven textures, closely aligning with the ground-truth defect zones identified by domain experts. Integrated Gradients produced smooth attribution maps concentrated around defect contours, suggesting that the classifier’s activations were driven by physically meaningful surface variations rather than background illumination or part geometry. The Saliency maps reinforced these findings by revealing high-gradient responses precisely at surface discontinuities, while Occlusion analysis confirmed that masking the defective region resulted in a substantial reduction in the model’s confidence score. Together, these interpretability outcomes validate that the YOLOv8n model’s decision-making process is both spatially focused and industrially relevant, making it suitable for deployment in automated visual inspection pipelines.

**Figure 11:**
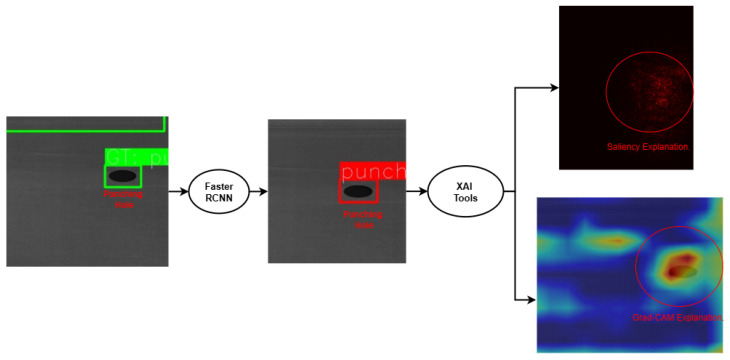
The Faster R-CNN model detects a punching hole defect, followed by XAI tools generating Saliency and Grad-CAM maps that highlight the regions influencing the model’s decision, improving transparency and human interpretability.

In the case of the metal surface defect detection dataset, the Faster R-CNN model was analysed to examine whether its predictions were accompanied by coherent spatial explanations. Grad-CAM heatmaps extracted from the network’s feature pyramid layers (Figure 11) revealed that the detector’s attention concentrated within annotated bounding boxes for diverse defect types, including scratches, dents, and surface inclusions. This alignment between the model’s activation patterns and the annotated ground truth underscores its capacity for interpretable localisation. Moreover, saliency visualisations provided finer delineation of defect boundaries, indicating that the detector relied on texture variations and edge discontinuities that are consistent with physical defect manifestations. Importantly, explainability also revealed potential limitations, such as partial attention on background reflections in high-gloss samples, highlighting the usefulness of XAI in diagnosing model biases and guiding further dataset refinement.

Collectively, the visual XAI analyses across both datasets demonstrate that deep learning models can produce interpretable, physically consistent justifications for their decisions when supported by appropriate explainability frameworks. Beyond confirming that models attend to relevant regions, these visual explanations empower human operators to audit AI reasoning, verify conformity with industrial standards such as EN4179, and confidently integrate AI systems into high-value manufacturing workflows.

### 7.2. Acoustic Anomaly Detection and Explainability

This section presents the experimental results and technical interpretation of the acoustic anomaly detection framework based on the Isolation Forest (IF) algorithm integrated with XAI tools. The study focuses on developing an interpretable, unsupervised learning pipeline capable of detecting abnormal sound events in industrial environments while maintaining full transparency of the model’s decision process. The results encompass feature extraction, model training, quantitative performance evaluation, and interpretability analysis, collectively demonstrating that the system achieves robust detection accuracy and high transparency—key requirements for trustworthy AI in manufacturing.

#### 7.2.1. Feature Extraction and Data Representation

Each audio sample was preprocessed at a fixed sampling frequency of 16 kHz and divided into overlapping frames of 1024 samples with a hop length of 512 samples. For each frame, seven time–frequency features were extracted using the librosa library, resulting in a seven-dimensional feature vector defined as:

$$f_{t}=\left[{\mathrm{RMS}}_{t},{\mathrm{ZCR}}_{t},{\mathrm{Centroid}}_{t},{\mathrm{Bandwidth}}_{t},{\mathrm{MFCC}1}_{t},{\mathrm{MFCC}2}_{t},{\mathrm{MFCC}3}_{t}\right],$$

where RMS denotes Root Mean Square energy, ZCR represents the Zero Crossing Rate, Centroid and Bandwidth correspond to the spectral centroid and bandwidth, and MFCC1–3 are the first three Mel-Frequency Cepstral Coefficients. These descriptors collectively capture amplitude, frequency, and timbral characteristics of the acoustic signal. To ensure numerical stability and uniform scaling across features, the data were standardised using z-score normalisation:

$$f_{t,i}^{\prime }=\frac{f_{t,i}-\mu_{i}}{\sigma_{i}},$$

where $\mu_{i}$ and $\sigma_{i}$ represent the mean and standard deviation of feature *i* computed from the training set.

#### 7.2.2. Model Training and Parameterisation

An Isolation Forest (IF) model was trained exclusively on feature vectors extracted from recordings of normal machine operation. This approach enables unsupervised learning of the statistical distribution of healthy system behavior. The model’s hyperparameters were configured as follows: number of estimators = 100, contamination = 0.05, and random state = 42 for reproducibility.

The IF algorithm isolates anomalies by recursively partitioning the feature space using randomly selected attributes and thresholds, yielding an anomaly score for each input vector:

$$s\left(f_{t}\right)=-h\left(f_{t}\right),$$

where $h(f_{t})$ denotes the average path length required to isolate $f_{t}$ within the ensemble of decision trees. Frames exhibiting higher anomaly scores are more likely to correspond to abnormal conditions.

#### 7.2.3. Threshold Selection and Frame-Level Detection

A global threshold θ was determined empirically to maximise the separation between normal and abnormal recordings. During inference, each test file was segmented into frames, and the anomaly score of each frame was compared against θ. Frame-level predictions were generated according to:

$$y_{t}=\left\{\begin{array}{cc} 1, & \mathrm{if}\;s(f_{t})>\theta ,\\ 0, & \mathrm{otherwise}. \end{array}\right.$$

The binary sequence $\left\{y_{t}\right\}$ was subsequently mapped to the time domain using the frame indices and hop length, allowing direct visualisation of abnormal regions on the waveform. Temporal overlays illustrate that the detected anomalies align with audible deviations such as abrupt impacts, irregular vibrations, or frequency drifts.

#### 7.2.4. Quantitative Evaluation

**Figure 12:**
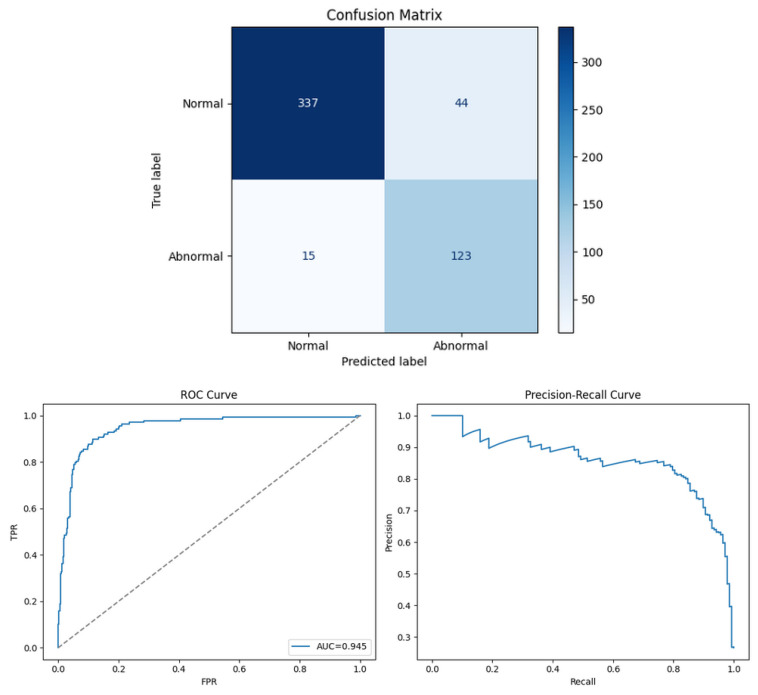
Performance evaluation of the Isolation Forest model for acoustic anomaly detection. The top panel shows the confusion matrix summarising classification accuracy across normal and abnormal classes, while the bottom panel illustrates the ROC and Precision–Recall curves, demonstrating strong discriminative capability (AUC = 0.945, overall accuracy = 89%).

The overall model performance is summarised in Figure 12, which presents the confusion matrix, Receiver Operating Characteristic (ROC) curve, and Precision–Recall (PR) curve for the test dataset. The model achieved an overall accuracy of 89%, with macro-averaged precision, recall, and F1-score of 0.85, 0.89, and 0.86, respectively. For the normal class, the precision and recall were 0.90 and 0.88, while for the abnormal class they were 0.74 and 0.89, respectively. The ROC curve exhibits an Area Under the Curve (AUC) of 0.945, confirming excellent discriminative ability between normal and abnormal operating conditions. The high recall for abnormal cases indicates strong sensitivity to fault events, which is crucial in predictive maintenance applications where missed detections can lead to costly system failures. Similarly, the PR curve demonstrates consistent precision at high recall levels, showing that the model maintains reliability under class imbalance.

These results confirm that the Isolation Forest, trained solely on normal data, effectively identifies unseen abnormal acoustic signatures without supervision, validating its suitability for real-time monitoring systems that demand both reliability and interpretability.

#### 7.2.5. Global and Local Explainability

To provide interpretability for the unsupervised model, the SHapley Additive exPlanations (SHAP) framework was employed. The scoring function for the SHAP analysis was defined such that higher SHAP values correspond to a higher likelihood of anomalous behaviour.

Score(X)=−IF.decision_function(X)

Global SHAP summaries aggregated at the dataset level revealed that RMS energy and Spectral Centroid exerted the strongest positive influence on anomaly scores, followed by MFCC1. These findings align with physical intuition—abnormal machine behavior typically manifests through increased vibration energy and shifts in spectral distribution.

Local interpretability was achieved through frame-specific waterfall plots, which decompose each frame’s anomaly score into additive feature contributions. In abnormal segments, elevated values of certain acoustic features contributed to higher anomaly scores, while lower values of other features reduced the anomaly response. Such analyses help reveal the specific acoustic characteristics that drive anomaly detection decisions.

#### 7.2.6. Signal-Level Summarisation

At the recording level, an anomaly ratio $R_{\mathrm{anomaly}}$ was computed as:

$$R_{\mathrm{anomaly}}=\frac{1}{T}\sum_{t=1}^{T}1\left\{s\left(f_{t}\right)>\theta \right\},$$

where *T* denotes the total number of frames. If $R_{\mathrm{anomaly}}$ exceeded 0.2, the corresponding signal was classified as abnormal. This simple yet effective rule allowed robust separation between healthy and faulty recordings, providing clear, interpretable outputs such as: “Signal classified as NORMAL (anomaly ratio = 0.13)” or “Signal classified as ABNORMAL (anomaly ratio = 0.75)”. Visualisation of detected anomalies confirmed that the IF model consistently highlighted time intervals associated with mechanical irregularities while maintaining low false-alarm rates in normal states.

#### 7.2.7. Case Study: Normal Signal (Normal_003)

**Figure 13:**
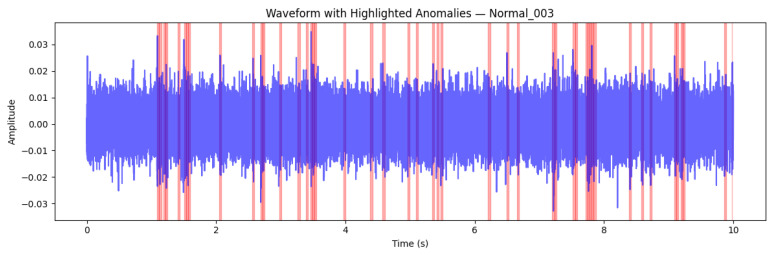
Waveform of the Normal_003 signal with anomaly spans highlighted in red. A total of 47 out of 313 frames (15.02%) were flagged by the Isolation Forest as locally anomalous. These sparse, short-duration events are non-contiguous and correspond to normal operational fluctuations, resulting in a final classification of “Normal”.

The Normal_003 recording represents a baseline example of healthy machine operation. The waveform shown in Figure 13 exhibits a stable amplitude profile with no sustained high-energy bursts or irregular transients. Although a small number of frames (47 out of 313, corresponding to $R_{\mathrm{anomaly}}=0.15$) were flagged as locally anomalous, these intervals were short, non-contiguous, and did not exceed the decision threshold at the signal level. This behaviour is consistent with minor acoustic fluctuations typical of normal mechanical processes such as background resonance, airflow variation, or sensor noise.

**Figure 14:**
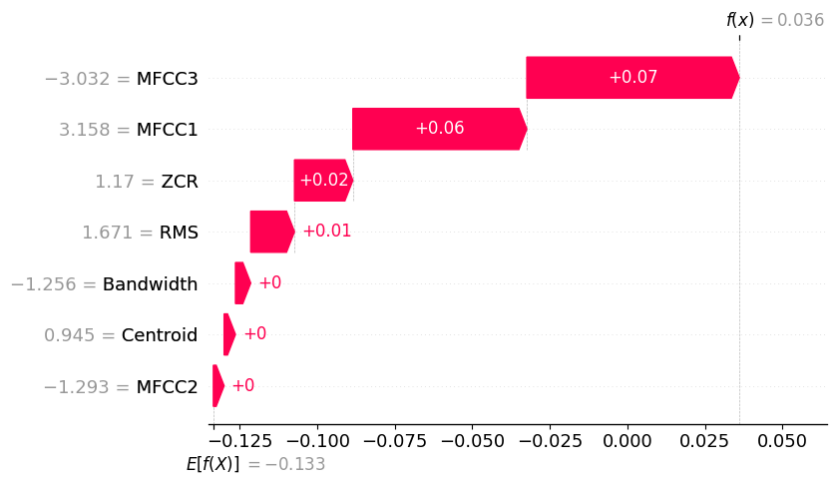
SHAP waterfall plot for a representative flagged frame in Normal_003. MFCC1 and MFCC3 contribute small positive values to the frame-level anomaly score, reflecting transient cepstral variability, while Bandwidth, Centroid, and MFCC2 provide compensating negative contributions. This balance keeps the aggregated anomaly score below the threshold, confirming normal behaviour.

From a statistical standpoint, the Isolation Forest’s decision function remained close to zero across most frames, indicating high similarity to the learned distribution of normal features. The corresponding SHAP-based feature attribution analysis further supports this conclusion. As illustrated in Figure 14, MFCC1 and MFCC3 provided small positive contributions to the local anomaly score, primarily reflecting transient cepstral shifts associated with momentary changes in acoustic tone. However, these effects were counterbalanced by negative contributions from Bandwidth, Centroid, and MFCC2, which stabilised the overall score and prevented escalation into an abnormal classification.

**Figure 15:**
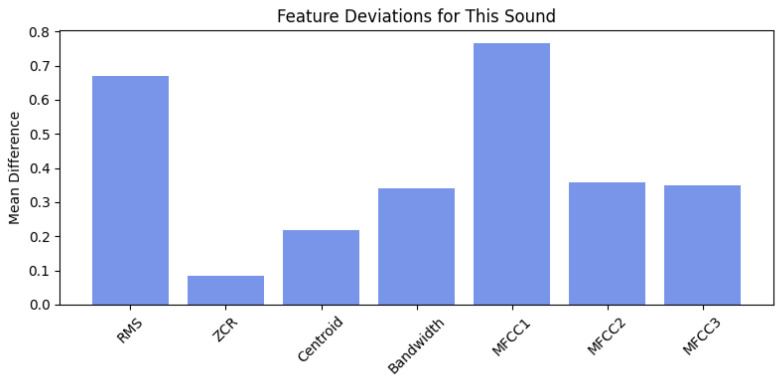
Per-sound feature deviation plot for Normal_003. The highest deviations are observed in MFCC1 (Δ=0.76) and RMS (Δ=0.67), followed by MFCC2 (Δ=0.36). All deviations remain within the statistical range observed for healthy signals, confirming stable machine operation.

The per-sound feature deviation analysis in Figure 15 quantifies this behaviour at the global level. The largest deviations from the mean normal baseline occurred in MFCC1 (Δ=0.76) and RMS energy (Δ=0.67), followed by MFCC2 (Δ=0.36). These moderate deltas indicate limited variation in spectral envelope and overall energy—expected for normal operation with mild acoustic modulation. Low deviations in Spectral Centroid and Bandwidth further confirm that the frequency distribution and spectral spread remained consistent with healthy operation.

From a physical interpretation perspective, these results indicate that the machine emitted a stable acoustic signature characterised by steady vibrational energy and a consistent harmonic content. The Isolation Forest’s decision to classify the recording as normal aligns with both the data-driven model behaviour and the underlying physics of the process: minor transient events may occur naturally, but they do not reflect a fault state or abnormal dynamics.

Overall, the Normal_003 case demonstrates the model’s ability to tolerate benign signal variability without overreacting to transient fluctuations. It validates the robustness and selectivity of the proposed anomaly detection framework—essential qualities for reliable industrial condition monitoring, where low false-alarm rates are critical for operator trust, maintenance efficiency, and compliance with explainable AI standards.

#### 7.2.8. Case Study: Abnormal Signal (Abnormal_004)

**Figure 16:**
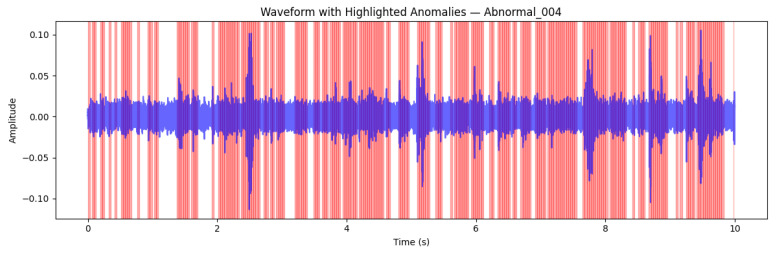
Waveform of the Abnormal_004 signal with anomaly spans highlighted in red. The dense and persistent red intervals across the 10 s duration reflect continuous abnormal behaviour. The high anomaly ratio ($R_{\mathrm{anomaly}}=0.74$) indicates that the majority of frames deviate from the learned distribution of normal acoustic features.

The Abnormal_004 recording represents a typical example of faulty machine behaviour. Unlike the stable characteristics of Normal_003, this signal exhibits sustained high-energy oscillations and irregular transient peaks, as seen in Figure 16. The Isolation Forest identified a large proportion of frames as anomalous, yielding an anomaly ratio of $R_{\mathrm{anomaly}}=0.74$, well above the decision threshold (θ=0.20). The dense red regions indicate that abnormal dynamics persisted across most of the 10 s recording, signifying a prolonged deviation from nominal machine operation.

**Figure 17:**
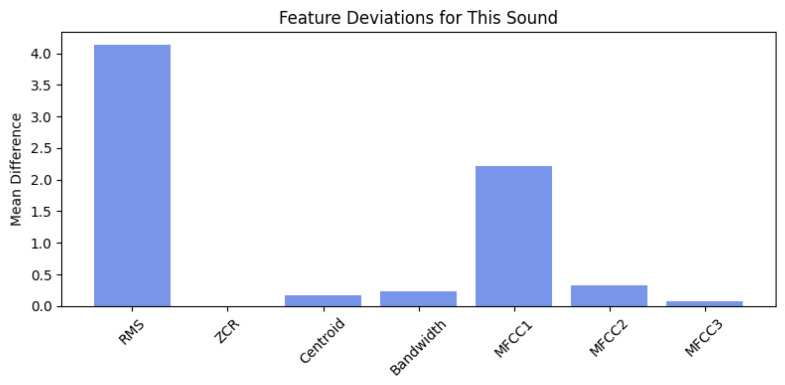
Feature deviation plot for Abnormal_004. RMS shows the largest deviation (Δ=4.1), followed by MFCC1 (Δ=2.2). These extreme deviations indicate high vibration energy and broad spectral modulation, characteristics consistent with mechanical faults.

From a statistical standpoint, the Isolation Forest’s decision function produced strongly negative scores across most frames, confirming a significant departure from the normal feature manifold. This is further supported by the per-sound feature deviation plot in Figure 17. Here, RMS energy exhibits an exceptionally large deviation (Δ=4.1), followed by MFCC1 (Δ=2.2) and MFCC2 (Δ=0.4). These large positive deviations indicate abnormal increases in vibration amplitude and cepstral coefficients, implying pronounced spectral and energetic shifts. Such deviations correspond physically to mechanical imbalance, frictional drag, or early-stage bearing impacts that alter both the energy envelope and the harmonic content of the signal.

**Figure 18:**
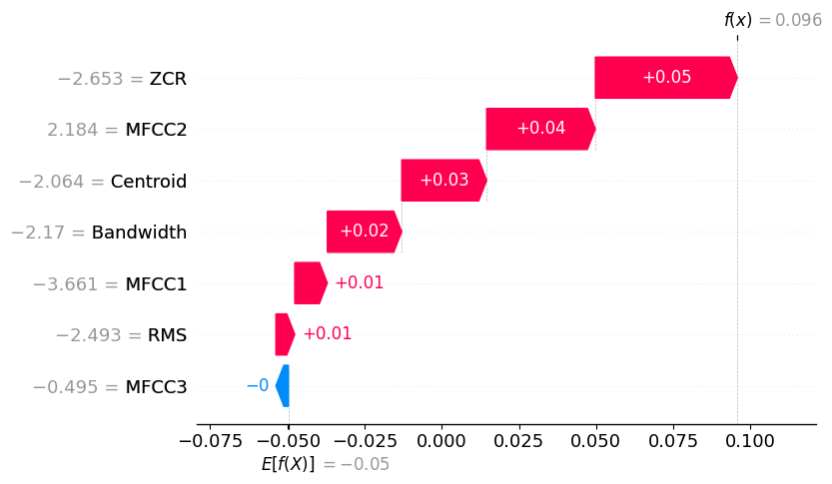
SHAP waterfall plot for a representative anomalous frame in Abnormal_004. RMS and MFCC1 dominate the positive contributions to the anomaly score, supported by moderate effects from MFCC2, Centroid, and Bandwidth. MFCC3 provides minimal negative contribution, indicating that the abnormality arises primarily from sustained increases in vibrational energy and cepstral dynamics.

Local feature attribution using SHAP analysis (Figure 18) provides additional interpretive insight. RMS and MFCC1 contributed the most substantial positive values to the frame-level anomaly score, clearly driving the Isolation Forest’s fault decision. MFCC2, Centroid, and Bandwidth also made positive contributions, indicating broader and more variable frequency content. By contrast, MFCC3 exerted a small negative influence, but insufficient to offset the dominant energy and spectral effects. The combined impact of these features yielded a net anomaly score of f(x)=0.096, reinforcing the classification of the frame—and by extension the entire recording—as abnormal.

From a physical perspective, the Abnormal_004 signal reflects a clear deterioration in mechanical stability. The elevated RMS levels denote excessive vibration energy, while the strong MFCC1 and MFCC2 activations suggest a deformation of the spectral envelope typically caused by rotating or oscillating components operating under mechanical stress. The temporal persistence of these deviations implies a structural rather than transient anomaly—such as bearing wear, imbalance, or partial shaft misalignment—leading to characteristic broadband noise and energy surges.

Overall, the Abnormal_004 case demonstrates the ability of the proposed framework to correctly identify and explain significant fault behaviour. The model not only detects the anomaly through statistical deviation but also attributes its cause to physically meaningful acoustic features. This strengthens the argument that the Isolation Forest—combined with SHAP-based interpretability—offers a robust, transparent, and physics-consistent tool for industrial fault detection and diagnosis.

#### 7.2.9. Discussion and Implications

The explainability analyses confirm that the Isolation Forest (IF) model grounds its predictions in physically meaningful acoustic features, rather than arbitrary statistical patterns. As observed across the analysed recordings, RMS energy and spectral centroid consistently act as dominant indicators of abnormal behavior, while MFCC coefficients capture subtle shifts in harmonic structure linked to machine health. High RMS values correspond to elevated vibrational energy, typically resulting from mechanical friction, bearing impacts, or shaft misalignment. Similarly, abrupt spectral centroid fluctuations reflect frequency-weighted energy shifts caused by unbalanced rotation or surface irregularities. By associating these measurable physical phenomena with model responses, the framework bridges the gap between data-driven detection and domain-grounded diagnosis, thus transforming the IF model from a black-box detector into an interpretable diagnostic tool.

Table 4 summarises the interpretability outcomes for all ten analysed signals. The per-signal analysis demonstrates the model’s capability to generalise across different acoustic signatures, accurately distinguishing between stable and degraded states. Normal recordings exhibit low anomaly ratios ($R_{\mathrm{anomaly}}<0.2$) and feature deviations limited to low-magnitude fluctuations in MFCC1, RMS, or MFCC2. In contrast, abnormal recordings display sustained anomaly ratios above 0.65 and amplified Δ values in RMS and MFCC1, confirming the strong association between high energy content and machine degradation. At the frame level, SHAP attributions reinforce this interpretation—normal cases show balanced contributions across features, whereas abnormal cases are dominated by energy-centric (RMS) and cepstral (MFCC1, MFCC2) components, highlighting the precise spectral–temporal signatures of mechanical faults.

These findings highlight the coherence between statistical and physical interpretability. The unsupervised IF model, trained solely on normal data, successfully learns the acoustic manifold of healthy machine states. When deviations occur, they correspond directly to physically plausible shifts in spectral energy or harmonic distribution—features that engineers can relate to tangible faults. This interpretive alignment provides strong evidence of model trustworthiness. Moreover, the quantitative explainability (via SHAP) ensures that operators can trace individual decisions back to specific features and time intervals, enabling fault localisation and root-cause inference.

From a system-design perspective, this integration of anomaly detection and SHAP-based interpretability strikes a crucial balance between accuracy, transparency, and deployability. The high AUC value of 0.945 confirms detection robustness, while per-signal interpretability mitigates the risk of overfitting and false alarms. The method’s unsupervised nature eliminates the dependency on labeled fault data—often unavailable in industrial environments—making it ideal for scalable, cross-asset condition monitoring.

In the context of Industry 4.0, such explainable anomaly detection systems play a pivotal role in achieving Trustworthy AI for predictive maintenance. They provide a dual advantage: reliable early fault detection and transparent decision rationale. This transparency not only fosters operator trust and regulatory compliance (e.g., AI Act, ISO/IEC 23894) but also supports continuous improvement loops where insights from SHAP-driven analysis can inform maintenance planning, sensor design, and process optimisation. Overall, the combination of Isolation Forest and SHAP represents a practical yet theoretically grounded approach toward interpretable, reliable, and data-efficient acoustic anomaly detection in manufacturing environments. In this study, systemisation refers to the repeatable integration and interpretation of explainability outputs across heterogeneous models and data modalities, rather than the definition of universal explainability methodologies or metrics. Building on the experimental findings, the final Section 8 concludes the paper by summarising key contributions and outlining future research directions to further advance trustworthy and explainable AI in smart manufacturing.

## 8. Conclusions and Future Work

This study has demonstrated how explainable artificial intelligence (XAI) can transform black-box models into transparent, auditable, and trustworthy tools for industrial decision-making. By applying XAI techniques to both vision-based and acoustic datasets, a cross-modal framework was developed that enhances human understanding of AI reasoning while maintaining strong detection performance.

In the visual domain, Grad-CAM, Integrated Gradients, and Occlusion analyses showed that YOLOv8n and Faster R-CNN models focused on physically meaningful defect regions, confirming interpretability in automated quality inspection. In the acoustic domain, SHAP analysis of Isolation Forest results provided feature-level transparency, revealing that anomalies were driven by energy and frequency deviations consistent with actual mechanical faults.

Explainability was found to complement accuracy rather than hinder it—enabling interpretable, compliant, and human-trusted AI systems within Industry 4.0 and the evolving Industry 5.0 paradigm. Moving forward, integrating Human-in-the-Loop (HITL) frameworks will be essential to strengthen collaboration between AI systems and human experts. Through interactive decision support, AI can assist humans in diagnosing faults and suggesting corrective actions, while human feedback refines AI reasoning and prevents automation bias.

The responsible integration of AI across all industries could be improved through the adoption of XAI principles, transforming opaque black-box systems into transparent frameworks. This transparency is critical for preventing cognitive offloading, preserving user memory, and mitigating the detrimental effects of over-reliance which erodes critical thinking and skill sets. By offering clear insight into why a decision was made, XAI helps to expose and eliminate systemic biases and also allows humans to learn from system outputs to build context and knowledge. This will promote innovation and improve the quality of work and decisions.

In a human-centric design, technology adopters should intentionally define the division of work to ensure the human role remains engaging and fulfilling. They should aim to actively support job retention, satisfaction, and preventing the human worker from being relegated only to tasks the AI cannot handle. AI presents an opportunity to use tools like gamification or accessible, customised explanations to make manufacturing jobs more motivating, enjoyable, and accessible to a more diverse talent pool. While research into human performance and experience is needed, the continued development in XAI is essential including the traceability of data sources. This extended view of XAI is vital to ensure systems are less biased and more inclusive, guaranteeing that the underlying data itself was obtained and utilised in a transparent and ethical way, which is key to building a truly collaborative and socially sustainable future.

Future work will advance toward causal and counterfactual explainability, multimodal data fusion, and embedding interpretability within digital twins. By fostering a symbiotic relationship between human expertise and machine intelligence, this research envisions AI systems that not only explain and predict but also collaborate with humans to make informed, transparent, and trustworthy industrial decisions.

## Figures and Tables

**Table 1:** Comparison of manufacturing data modalities and explainable AI use cases.

Data Domain/Type	Data Source	Example Use Cases	Task Type	Selected Dataset	Industrial Relevance	XAI Contribution
Vision-Based Image Classification (Grayscale/RGB Images)	Automated cameras, industrial vision sensors	Surface defect detection on castings, welds, and machined parts Paint and coating uniformity inspection Dimensional quality verification Porosity detection in additive manufacturing	Binary/Multi-Class Classification	Casting Dataset [17]	Represents automated visual inspection systems for defect and quality assurance in manufacturing	Provides visual interpretability using Grad-CAM, Integrated Gradients, and Occlusion to explain decision regions.
Vision-Based Object Detection and Localisation (Color Images with Bounding Boxes)	Industrial cameras, robotic inspection systems	Detection and localisation of cracks, scratches, and dents PCB solder-joint and component placement verification Tool wear and edge-chipping localisation Weld seam tracking and uniformity analysis	Multi-Class Object Detection	Defects Dataset [18]	Reflects industrial inspection tasks requiring classification and spatial localisation for defect identification	Improves model transparency via Grad-CAM and Saliency-based visualisation of spatial attention.
Acoustic/Vibration-Based Time-Series Analysis (Audio or Vibration Signals)	Microphones, accelerometers, vibration sensors	Machine condition monitoring (bearings, fans, motors, pumps) Tool chatter, imbalance, or looseness detection Predictive maintenance using sound and vibration data Leakage detection in pneumatic and compressor systems	Unsupervised Anomaly Detection	MIMII Dataset [19]	Models acoustic condition monitoring for early fault detection and predictive maintenance	Uses SHAP and LIME to explain feature-level reasoning in anomaly detection.

**Table 2:** Comparison of explainable AI adoption in manufacturing studies.

Capability	[33]	[34]	[35]	[36]	This Work
Applied to Predictive Maintenance	✓	✓	×	×	✓
Applied to Quality/Defect Analysis	×	×	✓	×	✓
Explicit XAI Technique Evaluation	✓	✓	✓	×	✓
Human-centred Interpretability Emphasis	×	✓	×	✓	✓
Multi-modal (vision + acoustic)	×	×	×	×	✓
Unsupervised + Supervised Integration	×	×	✓	×	✓
Systematic Evaluation Framework	×	×	×	×	✓

**Table 3:** Comparison of publicly available industrial datasets supporting trustworthy AI.

Dataset	Modality	Size	Labels	Task(s)	Industrial Domain
Casting Product Dataset [17]	Grayscale Images (300 × 300)	7348	Binary (OK vs. Defective)	Classification	Visual inspection of castings
GC10-DET [18]	Color Images with Bounding Boxes	2300 images, 2280 .xml labels	10 defect types with spatial location	Detection + Classification	Steel surface inspection
MIMII [19]	Acoustic Recordings	Several hours of audio	Normal vs. Abnormal	Anomaly Detection	Machine condition monitoring

**Table 4:** Per-signal interpretability summary showing anomaly ratios, dominant feature deviations, and SHAP-based contributors for Normal and Abnormal recordings.

Ref ID	File Name	Frames	Anom. Frames (%)	Threshold	Final Class	Top Δ Features (Δ)	SHAP Top Contributors
Normal_003	00000037.wav	313	47 (15.02)	−0.0873	Normal	MFCC1 (0.7647), RMS (0.6708), MFCC2 (0.3567)	MFCC1, MFCC3, ZCR, RMS
Normal_005	00000068.wav	313	78 (24.92)	−1.0873	Abnormal	MFCC1 (0.9427), MFCC3 (0.6974), ZCR (0.4648)	MFCC2, Centroid, ZCR
Normal_002	00000161.wav	313	42 (13.42)	−1.0873	Normal	Centroid (0.5619), ZCR (0.5589), MFCC3 (0.5482)	–
Normal_001	00000230.wav	313	204 (65.18)	−1.0873	Abnormal	ZCR (2.0425), MFCC2 (1.4328), Centroid (1.4098)	MFCC2, ZCR, Centroid
Normal_004	00000231.wav	313	46 (14.70)	−1.0873	Normal	Centroid (0.8532), MFCC2 (0.8267), ZCR (0.7526)	–
Abnormal_004	00000026.wav	313	207 (66.13)	−1.0873	Abnormal	RMS (4.1267), MFCC1 (2.2232), MFCC2 (0.3332)	MFCC2, Bandwidth, ZCR
Abnormal_005	00000029.wav	313	234 (74.76)	−1.0873	Abnormal	RMS (3.6100), MFCC1 (2.4282), MFCC3 (0.8650)	MFCC2, Bandwidth, Centroid
Abnormal_001	00000033.wav	313	253 (80.83)	−1.0873	Abnormal	RMS (3.7095), MFCC1 (2.6006), MFCC3 (0.3028)	MFCC2, Bandwidth, Centroid
Abnormal_003	00000053.wav	313	284 (90.73)	−1.0873	Abnormal	RMS (6.4281), MFCC1 (3.2028), Bandwidth (0.2648)	Bandwidth, MFCC2, Centroid
Abnormal_002	00000096.wav	313	247 (78.91)	−1.0873	Abnormal	MFCC2 (2.0744), Centroid (1.7369), Bandwidth (1.4135)	ZCR, MFCC3, Centroid

## Data Availability

The original contributions presented in this study are included in the article. Further inquiries can be directed to the corresponding author.

## Abbreviations

The following abbreviations are used in this manuscript:

AI	Artificial Intelligence
XAI	Explainable Artificial Intelligence
CNN	Convolutional Neural Network
Faster R-CNN	Faster Region-Based Convolutional Neural Network
YOLOv8	You Only Look Once, version 8
Grad-CAM	Gradient-weighted Class Activation Mapping
IG	Integrated Gradients
IF	Isolation Forest
SHAP	Shapley Additive Explanations
MFCC	Mel-Frequency Cepstral Coefficients
RMS	Root Mean Square
ZCR	Zero-Crossing Rate
ROC	Receiver Operating Characteristic
PR	Precision–Recall
AUC	Area Under the Curve
F1-score	Harmonic Mean of Precision and Recall
ISO/IEC	International Organization for Standardization
	/International Electrotechnical Commission
AI Act	European Union Artificial Intelligence Act
AMRC	Advanced Manufacturing Research Centre
HVMC	High-Value Manufacturing Catapult
RGB	Red–Green–Blue
